# Status of Household Solid Waste Management and Associated Factors in a Slum Community in Kampala, Uganda

**DOI:** 10.1155/2020/6807630

**Published:** 2020-05-06

**Authors:** Charles Ssemugabo, Solomon Tsebeni Wafula, Grace Biyinzika Lubega, Rawlance Ndejjo, Jimmy Osuret, Abdullah Ali Halage, David Musoke

**Affiliations:** Department of Disease Control and Environmental Health, School of Public Health, Makerere University College of Health Sciences, Kampala, Uganda

## Abstract

**Background:**

Only a third of the total waste generated in slum communities in Kampala is collected and disposed of to the landfill every month. This study assessed the status of household solid waste management and associated factors in a slum community in Kampala, Uganda.

**Methods:**

We conducted a community-based cross-sectional study involving 395 households using a semistructured questionnaire and an observational checklist. Proper solid waste management was determined based on possession of waste collection and storage receptacle; collection receptacle ability to minimise nuisances (covered); segregation of waste; presence of flies and other vectors; and collection receptacle fill status. Prevalence rate ratios and their 95% confidence intervals were used as a measure of association.

**Results:**

Only, 41.3% (163/395) of the households exhibited proper waste management practices. The majority of the households 85.8% (339/395) owned solid waste storage receptacles, most of which were sacs 61.7% (209/339) and would minimise nuisances 72.9% (245/339). The main type of waste collected was biodegradable materials 56.7% (224/395). The majority of the households 78.7% (311/395) did not segregate their waste. Solid waste was mainly transported to the collection point by pulling the collecting sac 54.4% (215/395). The city authority 73.9% (292/395) and private companies 12.9% (51/395) were the major entities collecting waste. Factors associated with proper waste management were collecting waste in plastic containers (adjusted PR = 1.27, 95% CI (1.04–1.55)), polythene bags (adjusted PR = 0.26, 95% CI (0.14–0.47)), and paper bags or metallic bins (adjusted PR = 0.13, 95% CI (0.03–0.44)) as well as awareness of solid waste management laws (adjusted PR = 1.49, 95% CI (1.20–1.85)) and the dangers of improper solid waste management (adjusted PR = 2.15, 95% CI (1.51–3.06)).

**Conclusion:**

Solid waste management was generally poor. As such, a cascade of interventions that address knowledge, physical, and behavioural aspects of solid waste management is required to improve its management in slum communities.

## 1. Introduction

Eight people died in low-lying slum communities in the outskirts of Kampala due to flash floods during the first rainy season of 2019 [1]. The flash floods were attributed to among others blockage of drainage channels with solid wastes. Many of the households in slum communities have been reported to indiscriminately manage their waste. The problem is likely to escalate with the estimated increase in population and consequently unplanned urbanization resulting in slum development in sub-Saharan Africa.

The generation of solid waste is indeed on the rise globally. Currently, cities around the world generate over 1.3 billion tonnes of waste annually, with this approximated to increase to 2.2 billion tonnes by 2025 [2]. This increase in the amount of solid waste generated is estimated to be much higher in developing countries due to rapid urbanization [3]. Today, Uganda is rapidly growing with annual urbanization and population growth rates of 5.1% and 3.3%, respectively [4]. However, the existing infrastructure for services such as solid waste management does not cope with the increased urbanization and waste generation [5]. Overall, approximately 28,000 tonnes of waste is collected in Kampala and delivered to the landfill every month, which accounts for only 40% of the total waste generated in the city [6]. The remainder of the waste generated is indiscriminately disposed of resulting in environmental and public health problems such as blockage of drainage channels and consequently flush floods. Other environmental health challenges due to poor solid waste management include pollution (water and soil) resulting in spread of diarrheal diseases [3].

Out of the 1,619,900 people that live in Kampala [4], approximately 53.6% (868,266) live in crowded and informal slum settlements, most of which are located in low-lying zones and wetlands (United Nations, 2014). This has resulted in overcrowding and development of more informal settlements. Although solid waste collection is a core service that should function well at community level, it has turned out to be a major challenge that slum residents, city authorities, and leaders are all grappling with [7]. Solid waste management involves control of waste generation, storage, collection, transfer and transport, processing and disposal of solid waste basing on best practices of public health, economics, and environmental consideration [8]. Lubaga division where Kasubi parish is located collects over 3,400 tonnes of solid waste per month [6]. However, a significant percentage of solid waste is dumped in unauthorized sites including drainage channels [6]. Moreover, there are only a few designated communal garbage collection points [7]. This is compounded by the fact that land lords are unwilling to give away a portion of their land for allocation to solid waste collection points, citing poor maintenance of waste collection sites [7, 9].

Kampala Capital City Authority (KCCA) is supporting collection of garbage generated at household level at a subsided fee. However, slum dwellers forfeit this service because of unaffordability, inaccessibility to the waste collection vehicles, disappointments from the solid waste collection companies, and ignorance of importance solid waste management services [10]. Previous studies have not employed observations to ascertain the actual status of solid waste management [10, 11], which this study employed. As such, the findings on solid waste management in this study are directly observed. This study therefore aimed at determining status of solid waste management and associated factors in households in a slum community in Kampala, Uganda.

## 2. Methods

### 2.1. Study Design

We carried out a community-based cross-sectional study using a semistructured questionnaire administered and an observational checklist collected data on solid waste management practices among slum households.

### 2.2. Study Area

The study was carried out in Kasubi Parish in Rubaga Division, Kampala. Kasubi comprises one of the largest slums located in the outskirts of Kampala. Kasubi Parish is comprised of largely informal and substandard housing and small scale businesses. It has a population of 384,386 people living in over nine zones [12]. Kasubi Parish has a high population density, uneven terrain, and poor sanitation and hygiene conditions and is in close proximity with the central business Center of Kampala. Thus, it often experiences challenges in managing solid waste especially at household level.

### 2.3. Sample Size and Sampling

Using the formulae for cross-sectional studies [13] and assuming an alpha of 0.05, power (1-beta) of 0.80, a sampling error of 5%, a nonresponse rate of 5%, and a statistically conservative prevalence of 50% for households that do not properly manage their solid waste, a final sample size of 401 households was obtained. The 50% prevalence of households which did not properly manage their waste was used to obtain an unbiased sample because previous studies carried out in this area were not focused on proper management of waste [14–16]. The sampling strategy that we used has been previously described [17]. Briefly, the sample size was distributed proportionately based on population size across the six selected out of the nine zones that make up Kasubi Parish. The number of households in each zone was obtained from Lubaga Division offices, and sampling proportionate to size was used to obtain the number of target households from each zone. Households, defined by the Uganda Bureau of Statistics (UBOS) as a group of persons who normally live and eat together [12], were selected using systematic random sampling. In each zone, the number of households was divided by the number of households to be selected to create a sampling interval. The first household in a zone was selected randomly, while subsequent households were selected by skipping a number of households equivalent to the sampling interval until the sampled number of households in that zone was achieved. Within the household, the household head or an adult above 18 years in the absence of the household head was interviewed.

### 2.4. Data Collection

Data were collected using a semistructured questionnaire and observational checklist. We asked respondents on whether they possessed solid waste containers, categories of waste collected, whether they segregated their waste, how they transported it to the collection point, distance to the nearest collection point, and the cost of data collection. Using an observational checklist, we observed the type of waste container households used, alternative containers to facilitate segregation, presence of files and any other vectors, and whether containers were full or not. The questionnaire was developed based on reviewed literature on solid waste management [6, 10, 18, 19]. Data collection tools were pretested in Mulago slum within the city which has similar characteristics with the study area. During pretesting, validity and reliability of individual questions in the questionnaire were assessed. Phrasing of some questions and their anticipated responses were revised to ensure generation of true responses. Trained Research Assistants who were Environmental Health students of Makerere University collected the data from all selected households.

### 2.5. Data Management and Analysis

Data were examined and cleaned on a daily basis during data collection and entered in EpiData version 3.02 (EpiData association; Denmark). To determine the status of household solid waste management (Outcome Variable), which was classified as proper or improper solid waste management, a score was generated from five questions which assessed the solid waste management practices at household level. These included (1) possession of solid waste management containers (yes/no); (2) solid waste management container which minimises nuisances (yes/no); solid waste management receptacle which minimises nuisances is a latent variable that was based on manifest variables, possession of solid waste storage receptacle, and type of solid waste storage receptacle used; the variable solid waste management container which minimises nuisances was defined as receptacle that was well covered and does not permit breeding of vectors such as insects and flies; (3) solid waste which was segregated (yes/no); (4) files and other vectors not seen around containers (yes/no); and (5) waste storage container not overfilled (yes/no). As described in previous studies, these questions highlight aspects of solid waste collection and storage at household level typical in a slum setting [20, 21]. Each response to the questions that emphasizes appropriate practice was assigned code 1 and the unsatisfactory practiced one was assigned code 0. In order to generate the cut-off for the outcome variable, we calculated the mean and median of the 3.045 and 3, respectively. The fact that the mean and median were similar demonstrated that our outcome variable is normally distributed. Since our outcome variable is categorical, the cut-off cannot contain a decimal point. Therefore, we decided to choose the nearby whole number above the mean and median which is 4 as out cut-off. Households that had a score of at least 4 out of 5 were classified as having proper practices and households with score of 3 or less were classified as having improper waste management practices. Other key variables to our study including awareness of waste management laws were measured by asking respondents whether they were aware of any laws govern management of solid waste in their area with responses yes and no. Knowledge on dangers of poor solid waste management was measured using two manifest variables (questions), that is, “do you know the dangers associated with poor solid waste management with responses yes and no” and “mention the dangers associated with poor solid waste management, with responses such as attraction of vectors and vermin, smells, unsightliness, accidents, and fire.” Any respondent who mentioned yes on knowledge of dangers of poorly managing solid waste but did not know the actual dangers was scored “0” for the latent variable, knowledge on the dangers of poor waste management. Using STATA version 14.0 (Stata Corp, Texas, USA), we analysed the data on proper waste management and associated factors. A generalized linear model of the Poisson family and log link with robust standard errors while applying a forward elimination method was used to generate prevalence rate (PR) ratios for measuring the association between the outcome and independent variables. Prevalence rate (PR) ratios were used since the outcome of interest was highly prevalent that is >10%, and odds ratios tend to overestimate the risk ratios in such circumstances [22, 23]. Simple models consisting of an outcome and a single independent variable were run to obtain the unadjusted PRs. In the multivariable model, variables which were significant at simple models (*p* < 0.05) were included while adjusting for age and sex. Stepwise backward elimination was applied until only significant variables and those that improved fit of the model were retained. The unadjusted and adjusted PRs and their corresponding 95% confidence intervals are presented. A *p*-value of less than 0.05 was considered for statistically significant associations.

### 2.6. Ethical Considerations

Ethical approval for the study was obtained from the Makerere University School of Public Health Higher Degrees, Research and Ethics Committee (101) and registered by the Uganda National Council for Science and Technology registration (HS 867). Participation in the study was voluntary and household heads provided written informed consent.

## 3. Results

### 3.1. Sociodemographic Characteristics of Participants

A total of 395 respondents participated in the study out of the 401 resulting in a response rate of 98.5%. Majority of the participants were females 75.9% (300/395), were Christians 77.5% (306/395), attained postprimary education 71.4% (282/395), and were aged 18–29 years 63.5% (250/395). Most of the participants 45.1% (178/395) were engaged in business (Table 1).

### 3.2. Solid Waste Management Practices

The majority of the households 85.8% (339/395) owned solid waste storage receptacles, most of which were sacs 61.7% (209/339) and minimising nuisances 72.9% (245/339) and were not overfilled 72.9% (247/339). The main types of waste collected were biodegradable materials (food remains and vegetation) 56.7% (224/395) and ashes and dust 29.4% (116/395). The majority of the households 78.7% (311/395) did not segregate their waste. Solid waste was mainly transported to the collection point by pulling the collecting sac 54.4% (215/395), carrying over the head 26.6% (105/395), and using wheel barrows 12.4% (49/395). The city authority 73.9% (292/395) and private companies 12.9% (51/395) were the major entities collecting waste. Only 32.9% (130/395) of the households paid for solid waste collection at a cost of less than 1 USD 93.1% (121/130). Most of the respondents were aware of the solid waste management laws 50.6% (200/395) and dangers of poor solid waste management 69.6% (275/395). Overall, 41.3% (163/395) of the households exhibited proper waste management practices (Table 2).

### 3.3. Factors Associated with Household Level Solid Waste Management Practices

Sex, age, education level, marital status, religion, and occupation of the household head were not significantly associated with their household's solid waste management status. Households whose main storage container were plastic bags were 1.3 times more likely to exhibit proper solid waste management practices compared to those that used sacks (adjusted PR = 1.27, 95% CI (1.04–1.55)). However, households whose main waste storage receptacle were polythene bags (adjusted PR = 0.26, 95% CI (0.14–0.47)) or other receptacles (paper bags and metallic bins) (adjusted PR = 0.13, 95% CI (0.03–0.49)) were 74% and 87%, respectively, less likely to exhibit proper solid waste management practices than those who used sacks. Household heads who were aware of solid waste management laws (adjusted PR = 1.49, 95% CI (1.20–1.85)) were 1.5 times more likely to exhibit proper waste management as compared to those who were not aware. Household heads who knew the dangers of poor solid waste management (adjusted PR = 2.15, 95% CI (1.51–3.09)) were 2.2 times more likely to practice proper solid waste management as compared to those who did not know (Table 3).

## 4. Discussion

This study sought to understand the status of household solid waste management and associated factors in a slum community in Kampala, Uganda. Our findings show that biodegradable waste was the major type of waste collected. Majority of the households possessed solid waste collection receptacles with sacks as the most used storage receptacle. Few households segregated and paid for collection of their solid waste. Less than half of households exhibited proper solid waste management practices. Households that used plastic containers for waste collection and household heads that were aware of the solid waste management laws and the dangers of improper solid waste management were more likely to properly manage their waste. On the contrary, households that used polythene bags, paper bags, or metallic containers were less likely to practice proper waste management practices. This implies that knowledge and solid waste management utilities are very pertinent to proper management of solid waste in less resourced households.

Biodegradable wastes such as food remains and vegetation were the major form of solid waste generated by households. This is understandable as the slum community is largely a residential area, as such biodegradable waste from the kitchen is often generated. Our findings are similar to those of studies carried out in other parts of Uganda [10, 11, 24, 25]. However, it is clear from our findings that few households engaged in segregation of biodegradable waste from other types. Our findings are similar to those from studies in Ghana, Ethiopia, and Kenya where a small percentage of households segregated their waste [18, 19, 26]. Projects implemented in slum communities sensitize households on solid waste management especially separation at the point of practice [27, 28], but many households may not afford multiple receptacles required to segregate waste [29]. Unlike our research, a related study found that a relatively high number of households were willing to engage in waste segregation [10] and in another, half of the respondents segregated their waste [11]. Solid waste segregation allows further processing and value extraction through recycling and composting [30]. However, in urban areas most residents do not engage in such processing including compositing attributed partly to absence of a garden [11]. It is important that solid waste management initiatives in slums promote separation of waste at household level so as to facilitate proper solid waste management.

Most households in our study used sacks to collect and store their solid waste. Sacks allow for storage of more wastes for a relatively long period of time compared to other household solid waste collection receptacles such as plastic containers and polythene bags. In addition, sacs are also relatively cheap compared to the plastic containers. However, sacs are not a suitable solid waste receptacle storage as they are nonabsorbent, noncombustible, and not watertight as prescribed by the Kampala solid waste ordinance of 2000 [31]. Relatedly, a similar study reported polythene bags, a substandard waste storage receptacle as the main storage receptacle used in two slums in Uganda [10]. This implies that waste collection in slums is conducted using substandard receptacles that could lead to nuisances around households. Therefore, waste management programmes in slums should encourage use of standard containers for waste collection.

Majority of households did not pay for collection of their solid waste. This implies that their waste might not have been collected by KCCA or other private companies that are involved in waste collection. So, these households were possibly disposing their waste indiscriminately. On the contrary, studies carried out in Nepal, Nigeria, India, and Ethiopia showed that urban households were willing to pay more for proper waste management [32–36]. Indeed, urban residents in Nepal, Nigeria, and Ethiopia where urban residents were paying 0.74, 2, and 1.07 USDs, respectively, for waste collection [33, 34, 36]. Unlike our study, these studies were carried out in more resourced communities. Our study also found that almost all household that paid for waste collection incurred less than 1 United States of American dollar (USD) per collection. Authorities should increase access to solid waste management services and encourage households to utilize them.

Households in our study that used plastic containers were more likely to properly manage their waste compared to those that used sacs. This is understandable as plastic containers usually have tight covering and eliminate nuisances unlike sacs. Covered plastic bins protect the waste from direct exposure to vectors and vermin, as well as mitigate bad odour and unsightedness consequently contributing to proper waste management [37, 38]. It is also possible that households that could afford plastic containers could also afford to pay waste collection fees furthering proper waste management practices. Indeed, in our study, use of substandard waste collection receptacles is corroborated with improper management of solid waste with households that used polythene and paper bags more likely to improperly manage their waste. Our finding are similar to those from a Ghanaian study which found that indiscriminate dumping of waste was practiced by households that used polythene bags [18]. This demonstrates that waste collection and storage receptacles are very instrumental in ensuring proper waste management and so should be availed at household level within slums.

Among respondents, awareness of dangers of poor waste management and laws that govern management of waste were associated with proper waste management practices. Awareness of the dangers of poor waste management and existing laws and associated penalties increases individual's perceived susceptibility associated diseases and penalties and hence motivates them to adopt appropriate practices [39]. In fact, indiscriminate waste management has been associated with health challenges such as pollution of water and air as well as breeding of vectors among others [40–42]. Our findings are similar to those in studies carried out in Kenya and Guinea that highlighted lack of awareness of proper waste management practices as well as laws and policies as drivers of poor waste management [43, 44]. Conversely, other studies have argued that awareness of legislation or good practices alone does not result into proper solid waste management practices but must be followed with strict enforcement [45, 46]. So, there is need for solid waste management promotion initiatives to boost slum dwellers' knowledge on associated health risks and penalties in order to facilitate proper solid waste management.

Although some practices were self-reported, status of solid waste management was assessed through observation of waste management receptacles and practices. Given the similarities in slums settings in Kampala, our findings could be generalized to the entire slum community within Kampala. In addition, the study makes a significant contribution to the understanding of waste management practices in slums which have not be well studied especially the use of observations. Although face validity and reliability of the questionnaire were carried out during pretesting, it was not done for individual questions.

## 5. Conclusion

Proper solid waste management was generally low with majority of households using sacks as their waste storage receptacles and not segregating their waste. Use of plastic containers, awareness of waste management laws, and danger of poor waste management were associated with exhibition of proper management practices, while use of substandard receptacles like polythene and paper bags was associated with improper waste management practices. Therefore, in order to improve the solid waste management in slum household, there is need to employ a cascade of interventions that address the knowledge, physical, and behavioural aspects of solid waste management.

## Figures and Tables

**Table 1:** Sociodemographic characteristics of participants.

Characteristics	Frequency (*n* = 395)	Percentage (%)
*Sex*		
Female	300	75.9
Male	95	24.1

*Age in years [mean (±SD)]*	30.0 (±10.8)	
14–29	250	63.5
30–45	104	26.4
46 and above	40	10.1

*Education level*		
None	27	6.8
Primary	86	21.8
Secondary	174	44.1
Tertiary	108	27.3

*Marital status*		
Single	84	21.3
Married	256	64.8
Widowed/separated/divorced	55	13.9

*Religion*		
Christian	306	77.5
Muslim	89	22.5
*Occupation*		
Business	178	45.1
Casual labour	57	14.4
Formal employment	72	18.2
Student	41	10.4
Others	47	11.9

**Table 2:** Solid waste management practices at household level.

Variables	Frequency (*n* = 395)	Percentage (%)
*Possession of a solid waste storage container* ^*∗*^ * (n* *=* *395)*		
Yes	339	85.8
No	56	14.2

*Type of solid waste storage receptacle used (n* *=* *339)*		
Plastic bin	36	10.6
Sacs	209	61.7
Polythene bags	63	18.6
Other containers like paper bags and metallic bins	31	9.1

*Solid waste storage container which minimises nuisances* ^*∗*^ * (n* *=* *339)*		
Yes	245	72.9
No	94	27.1

*Waste storage containers not overfilled* ^*∗*^ * (n* *=* *339)*		
Yes	247	72.9
No	92	27.1

*Flies and other vectors not seen around the container* ^*∗*^ * (n* *=* *339)*		
Yes	220	64.9
No	119	35.1

*Categories of wastes generated (n* *=* *395)*		
Biodegradable materials (food remains and vegetation)	224	56.7
Ashes and dust	116	29.4
Paper and cardboard	22	5.6
Plastics	10	2.5
Unclassified debris like wood/leather	23	5.8

*Solid waste was segregated* ^*∗*^ * (n* *=* *395)*		
Yes	84	21.3
No	311	78.7

*Transportation of solid waste to collection point (n* *=* *395)*		
On the head	105	26.6
Wheel barrow	49	12.4
Truck	26	6.6
Pulling the sac	215	54.4

*Distance to the nearest collection site (metres) (n* *=* *375)*		
<10	238	63.5
10–20	49	13.1
20–30	31	8.3
>30	57	15.2

*Entity collecting waste (n* *=* *395)*		
City authority	292	73.9
Private companies	51	12.9
Not collected	28	7.1
Do not know	24	6.1

*Paid for solid waste collection (n* *=* *395)*		
Yes	130	32.9
No	265	67.1

*Cost of solid waste collection in a week (n* *=* *130)*		
Less than 1 United States dollars (USD)	121	93.1
Above 1 USD	09	6.9

*Aware of waste management laws*		
Yes	195	49.4
No	200	50.6

*Knew the dangers of poor solid waste management*		
Yes	275	69.6
No	120	30.4

*Status of household solid waste management (n* *=* *395)*		
Improper	232	58.7
Proper	163	41.3

^*∗*^Variables used in calculating scores on the practices on solid waste management at households.

**Table 3:** Factors associated with household level solid waste management practices.

Characteristic	Proper solid waste management		Crude PR (CI)	*p*-value	Adjusted PR (CI)	*p*-value
	Yes *n* (%)	No *n* (%)				
*Sex*						
Male	45 (47.4)	50(52.6)	1			
Female	118 (39.3)	182(60.7)	0.83 (0.64–1.07)	0.153		

*Age group*						
14–29	102 (40.8)	148 (59.2.0)	1			
30–45	43 (41.3)	61(58.7)	1.01 (0.77–1.33)	0.924		
46 and above	18 (45.0)	22 (55.0)	1.10 (0.76–1.60)	0.608		

*Religion*						
Christians	122 (39.9)	184 (60.1)	1			
Muslim	41 (46.1)	48 (53.9)	1.16 (0.89–1.50)	0.283		

*Education level*						
Primary	36 (41.9)	50 (58.1)	1			
None	10(37.0)	17 (63.0)	0.88 (0.51–1.54)	0.664		
Secondary	79 (45.4)	95 (54.6)	1.08 (0.81–1.46)	0.593		
Tertiary	38 (35.2)	70 (64.8)	0.84 (0.59–1.20)	0.341		

*Marital status*						
Single	38 (45.2)	46 (54.8)	1			
Married	102(39.8)	154 (60.2)	0.88 (0.67–1.16)	0.374		
Widowed/divorced/separated	23 (41.8)	32 (58.2)	0.92 (0.63–1.37)	0.694		

*Occupation*						
Business	78 (43.8)	100(56.1)	1			
Formal employment	28 (38.9)	44 (61.1)	0.89 (0.64–1.24)	0.484		
Casual labour	23 (40.4)	34 (59.7)	0.92 (0.64–1.32)	0.651		
Student	17 (41.5)	24 (58.5)	0.95 (0.63–1.41)	0.787		
Other occupations	17 (36.2)	30 (63.8)	0.83 (0.54–1.25)	0.365		

*Solid waste storage receptacle type*						
Sacks	125 (59.8)	84 (40.1)	1		1	
Plastic bin	27 (75.0)	09 (25.0)	1.25 (1.01–1.56)	0.043	1.27 (1.04–1.55)	0.020^*∗*^
Polythene bags	09 (14.3)	54 (85.7)	0.24 (0.13–0.44)	<0.001^*∗*^	0.26 (0.14–0.47)	<0.001^*∗*^
Other containers (paper bags and metallic bins)	02 (6.4)	29 (93.6)	0.11 (0.03–0.41)	>0.001	0.13 (0.03–0.49)	<0.003

*Distance to the nearest collection point (metres)*						
<10	96 (40.3)	142 (59.7)	1			
10–20	25 (51.0)	24 (49.0)	1.26 (0.92–1.73)	0.144		
20–30	15 (48.4)	16 (51.6)	1.20 (0.81–1.78)	0.367		
>30	24 (42.1)	33 (57.9)	1.04 (0.74–1.47)	0.806		

*Mode of transportation used to carry waste to collection point*						
On the head	40 (38.1)	65 (61.9)	1			
Wheel barrow	25 (51.0)	24 (49.0)	1.34 (0.93–1.93)	0.119		
Truck	06 (23.1)	20 (76.9)	0.61 (0.29–1.27)	0.187		
Pulling the sack	92 (42.8)	123 (57.2)	1.12 (0.84–1.50)	0.431		

*Paid for solid waste collection*						
No	100 (37.7)	165 (62.3)	1			
Yes	63 (48.5)	67 (51.5)	1.28 (1.01–1.63)	0.037^*∗*^	0.95 (0.80–1.13)	0.598

*Aware of waste management laws*						
Not aware	56 (28.0)	144 (72.0)	1			
Aware	107 (54.9)	88 (54.9)	1.96 (1.52–2.53)	<0.001^*∗*^	1.49 (1.20–1.85)	<0.001^*∗*^

*Knew dangers of poor management of waste*						
No	144 (72.0)	56 (28.0)	1			
Yes	107 (54.9)	88 (45.1)	2.95 (1.97–4.43)	<0.001^*∗*^	2.15 (1.50–3.09)	<0.001^*∗*^

PR = prevalence rate ratio, CI = confidence interval, ^*∗*^*p* < 0.05, and level of confidence = 95%.

## Data Availability

The dataset used during the study is available from the corresponding author upon reasonable request.
